# Work-related quality of life in professionals involved in pediatric palliative care: a repeated cross-sectional comparative effectiveness study

**DOI:** 10.1177/26323524241247857

**Published:** 2024-05-09

**Authors:** Anne-Kathrin Gerber, Ursula Feuz, Karin Zimmermann, Stefan Mitterer, Michael Simon, Nicolas von der Weid, Eva Bergsträsser

**Affiliations:** Institute of Nursing Science, University of Basel, Basel, Switzerland; Institute of Nursing Science, University of Basel, Basel, Switzerland; Division of Pediatric Emergency Medicine, Department of Pediatrics, Inselspital, Bern University Hospital, University of Bern, Bern, Switzerland; Institute of Nursing Science, University of Basel, Bernoullistrasse 28, Basel 4056, Switzerland; Division of Pediatric Palliative Care and Children’s Research Center, University Children’s Hospital Zurich, Zurich, Switzerland; Institute of Nursing Science, University of Basel, Basel, Switzerland; Institute of Nursing Science, University of Basel, Basel, Switzerland; Division of Haematology–Oncology, University Children’s Hospital beider Basel (UKBB), Basel, Switzerland; Division of Pediatric Palliative Care and Children’s Research Center, University Children’s Hospital Zurich, Zurich, Switzerland

**Keywords:** compassion fatigue, compassion satisfaction, health personnel, palliative care, pediatrics, quality of life

## Abstract

**Background:**

Working in pediatric palliative care (PPC) impacts healthcare and allied professionals’ work-related quality of life (QoL). Professionals who lack specific PPC training but who regularly provide services to the affected children have articulated their need for support from specialized PPC (SPPC) teams.

**Objectives:**

This study had two objectives: (1) to evaluate whether the availability of a SPPC team impacted the work-related QoL of professionals not specialized in PPC; and (2) to explore the work-related QoL of professionals working in PPC without specialized training.

**Design:**

Repeated cross-sectional comparative effectiveness design.

**Methods:**

One hospital with an established SPPC program and affiliated institutions provided the intervention group (IG). Three hospitals and affiliated institutions where generalist PPC was offered provided the comparison group (CG). Data were collected by paper-pencil questionnaire in 2021 and 2022. The Professional Quality of Life (ProQOL 5) questionnaire was used to assess work-related QoL, yielding separate scores for burnout (BO), secondary traumatic stress (STS) and compassion satisfaction (CS). A descriptive statistical analysis was performed and general estimation equations were modelled. To increase the comparability of the IG and CG, participants were matched by propensity scores.

**Results:**

The 301 participating non-PPC-specialized professionals had overall low to moderate levels of BO and STS and moderate to high levels of CS. However, none of these scores (BO: *p* = 0.36; STS: *p* = 0.20; CS: *p* = 0.65) correlated significantly with support from an SPPC team. Compared to nurses, physicians showed higher levels of BO (1.70; *p* = 0.02) and STS (2.69; *p* ⩽ 0.001).

**Conclusion:**

Although the study sample’s overall work-related QoL was satisfactory, it showed a considerable proportion of moderate BO and STS, as well as moderate CS. To provide tailored support to professionals working in PPC, evidence regarding key SPPC support elements and their effectiveness is needed.

**Trial registration:**

ClinicalTrials.gov ID, NCT04236180.

## Plain language summary

Work-related quality of life in professionals involved in pediatric palliative care

- Why was this study done? Caring for children suffering from life-limiting conditions and their families impacts professionals’ work-related Quality of Life (QoL). Professionals without specific training often provide pediatric palliative care (PPC) to children and their families.

- What did the researchers do? We aimed to determine whether the work-related the QoL of professionals without specialised PPC training would be positively influenced when they were supported by PPC specialists. We also wanted to explore what person-specific factors might correspond with higher or lower work-related QoL. Work-related QoL was analysed in relation to burnout (BO), secondary traumatic stress (STS), and compassion satisfaction (CS). These variables’ levels were assessed with a questionnaire survey in 2021 and 2022.

- What did the researchers find? The 301 participating professionals had overall low to moderate levels of BO and STS and moderate to high levels of CS. There was no substantial difference in work-related QoL in the professionals supported by PPC specialists compared to those who did not receive specialist support. Physicians showed higher levels of BO and STS than nurses.

- What do the findings mean? Although the studied professionals’ overall work-related QoL was satisfactory, there is a considerable proportion of moderate BO and STS scores in professionals working with children suffering from life-limiting conditions. Further research should explore the specific needs of professionals not specialised in PPC.

## Introduction

Worldwide, an estimated 8 million children potentially need pediatric palliative care (PPC) due to life-limiting conditions;^1^ and that number is rising.^2,3^ Based on the UK prevalence of 66.4 per 10,000 population (aged 0–19 years), Switzerland is home to approximately 10,000 children with life-limiting conditions.^2,4^ A condition is considered life-limiting if there is no realistic hope of a cure or if the available treatments are likely to fail,^5^ encompassing a wide range of conditions and diseases.^6^

Children with life-limiting conditions commonly have high care needs, in which case they may benefit from PPC.^7^ The International Association for Hospice and Palliative Care defines Palliative Care as ‘the active holistic care of individuals across all ages with serious health-related suffering due to severe illness and especially of those near the end of life. It aims to improve the quality of life (QoL) of patients, their families and their caregivers’ (p. 761).^8^ Physical, developmental, psychological, ethical, spiritual and relational phenomena unique to children and their families are central to PPC.^9^ Furthermore, palliative care is not limited to end of life (EoL) care but can be provided alongside active curative treatment and throughout disease progression.^10^ Therefore, PPC should be regarded as an acceptable standard of care for children with life-limiting conditions.^11^

To address these children’s and their families’ multifaceted needs, they should be cared for by a specialized and dedicated multidisciplinary team.^12^ Specialized PPC (SPPC) is often provided through a hospital-based consultative service model.^13^ The consultative model entails direct clinical care for patients and their families and offers support and expertise to primary care teams in- and outside the hospital.^14^ Services provided by SPPC teams include symptom management, communication facilitation, shared decision-making and advance care planning and assistance with logistics or coordination of care, including transition to home and bereavement care.^13^

Working in pediatric care is demanding and comprises numerous aspects linked to increased emotional burden. There is a high potential for empathetic engagement, and relationships with families are inherently complex.^15^ In PPC, regular exposure to suffering and death intensifies the emotional burden.^11,16–18^ Emotional burden has been described as ‘emotional hazards’,^19^ ‘emotional cost’,^20^ ‘high emotional load’,^21^ ‘personal pain’^22^ and ‘labour in the sense of hard work’.^23^ As working in PPC influences the involved professionals’ work-related QoL in potentially negative ways, it also influences their ability to provide high-quality care.^24^ However, alongside these negative effects, working in PPC can be rewarding: even in such challenging situations, professionals can experience satisfaction.^25,26^

Work-related QoL is the ‘quality one feels in relation to their work as a helper’.^27^ (p. 8). Its positive and negative aspects have been conceptualized respectively through compassion satisfaction (CS) and compassion fatigue (CF). Burnout (BO) and secondary traumatic stress (STS) can be seen as aftereffects of CF.^27^ To date, few studies have analysed professionals’ work-related QoL in the context of PPC; as a result, empirical evidence on the topic is limited.^28^ A cross-sectional pilot study in PPC providers in the United States found that ‘distress about a clinical situation’, ‘emotional depletion’, ‘recent involvement in a clinical situation in which life-prolonging activities were not introduced’ and ‘not discussing distressing issues’^29^ all function as predictors for high CF, BO and low CS levels. Examining traumatic stress in PPC providers, MT Rourke inferred that PPC professionals are ‘trauma workers’,^28^ noting that their CF levels exceed the average levels in trauma and non-pediatric healthcare workers.^28^

To help professionals involved in PPC reduce their emotional burden and improve their work-related QoL, SPPC services may provide vital support. Studies have shown that non-specialized professionals wish for training and support from SPPC teams.^17,30^ In fact, the American Academy of Pediatrics considers such assistance so important – and the need for it so clear – that their policy specifies that experienced SPPC teams should provide it proactively.^31^ Today, professionals commonly provide PPC without specialized training. As a result, nurses, for example, report being unprepared, having limited expertise and feeling uncomfortable caring for a child in a palliative or EoL situation.^32^

Given the rapid development of SPPC programs and their potential impact, evaluating their effectiveness has become a priority of several research agendas.^33–35^ This includes investigating the effect of SPPC programs on non-specialized professionals involved in PPC. The purpose of this study was to evaluate whether the availability of an SPPC team would impact the work-related QoL of professionals not specialized in PPC and to explore the work-related QoL of professionals working in PPC without specialized training. To fulfil this purpose, we planned three tasks; (1) to measure work-related QoL in professionals not specialized in PPC; (2) to assess differences in the work-related QoL of professionals not specialized in PPC with and without the availability of an SPPC team; and (3) to explore which factors influence work-related QoL in professionals not specialized in PPC.

## Materials and methods

### Context, study design and setting

This study is part of the SPhAERA (Specialized Pediatric PAlliativE CaRe: Assessing family, healthcare professional and health system outcomes in a multi-site context of various care settings) research project to evaluate the effectiveness of an SPPC program by assessing clinical, service and economic outcomes. The comparative effectiveness study presented here applied a non-randomized, repeated cross-sectional design, collecting data at two time points separated by an interval of 1 year. The SPhAERA study protocol is published elsewhere.^36^

The study was conducted in 2021 and 2022 at three university children’s hospitals, one cantonal children’s hospital, and affiliated external institutions (e.g. home care services, long-term care institutions, pediatricians) in the German-speaking part of Switzerland.

The SPhAERA study was approved by the responsible ethics committees (BASEC Nr. 2019-01170), and complies with the Swiss Federal Act on research involving human beings. All invited professionals were provided with an information sheet and consent form to make an informed decision regarding their participation.

The reporting of this study conforms to the Strengthening the Reporting of Observational Studies in Epidemiology (STROBE) statement and follows the STROBE checklist for cross-sectional studies.^37^ The STROBE checklist for cross-sectional studies is provided as Supplemental Material.

### Study participants and recruitment

This study’s target population comprised professionals working in pediatrics without SPPC training. Eligibility criteria were defined to enrol those exposed to PPC, that is, participants needed to meet at least one of the following criteria within the last 12 months: (1) having cared for patients and their families in the palliative care phase over a minimum of 10 shifts (day/late shift) or consultations; and/or (2) having cared for a minimum of two patients and their families either in the EoL-phase or after the death of the child. The research team did not provide definitions for ‘palliative care phase’ or ‘EoL-phase’. Participants could judge at their discretion whether they deemed patients to be in the palliative care or EoL-phase. Professionals from the following groups were eligible: physicians, registered nurses, healthcare assistants, psychologists, social workers, physical therapists, occupational therapists, nutritionists, pastoral workers, logopedists, social pedagogues, remedial teachers and certified social care workers. In addition, they had to have been employed in their institution for at least 3 months and have passed their probation period. Professionals specializing in PPC and working in dedicated SPPC settings were excluded from study participation.

Recruitment took place in 2021 and 2022. The research team and local study coordinators contacted all professionals with direct patient contact at the four participating hospitals and potential participants from affiliated institutions. Each received an invitation letter *via* internal post or e-mail describing the study and its eligibility criteria and requesting their written informed consent if they wished to participate. For participants recruited in 2021, eligibility was reassessed for their participation in 2022.

### Intervention and comparison groups

The intervention group (IG) consisted of professionals working in one university children’s hospital with an established SPPC program that had been running for over 10 years. Eligible professionals from external institutions who were collaborating with this university hospital through the SPPC program and who wished to participate were also allocated to the IG. The SPPC program, that is, the intervention, was run by a multi-professional team including 12 members whose combined employment percentages equalled 5.7 full-time equivalents. The SPPC team offered direct care (e.g. care provided to the patient and/or their family), including medical, nursing, social, psychological and spiritual support during the palliative and bereavement phases. Additionally, indirect care and support (e.g. training and support provided to the primary care team) are integral to the SPPC program. SPPC services were offered 24/7 in- and outside of the hospital. These were provided by professionals specialized in PPC and working in a dedicated SPPC setting.^10^ In 2021 and 2022, the SPPC team cared respectively for 170 and 180 children and their families. Roughly 60–70 patients are newly admitted into the SPPC program every year.

**Figure 1.**
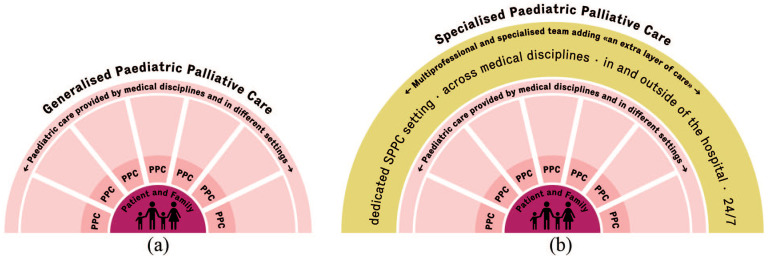
PPC *versus* SPPC. (a) Generalized PPC is provided by specialists of a given discipline and setting in a fragmented manner, as displayed by the separate pieces. Professionals engaging in generalized PPC have basic PPC training and experience without working fully engaged and dedicated in PPC. (b) SPPC is provided by professionals specialized in and working dedicatedly in PPC. SPPC teams are composed of professionals with various backgrounds, for example, medicine, nursing, psychology and are characterized by providing interprofessional consultative services, adding an extra layer of care. PPC, pediatric palliative care; SPPC, specialized pediatric palliative care.

The comparison group (CG) comprised professionals from the other three hospitals, including two university hospitals, a cantonal children’s hospital and external institutions affiliated with the CG hospitals. PPC was provided on a generalist level in the hospitals from the CG with hospital-based PPC teams and individual specialists present, but not specifically working in and dedicated to SPPC.^10^ The PPC teams in the CG hospitals were composed of physicians, nurses and, in one hospital, a psychologist. Overall, the three teams’ employment percentages equalled 2.9 full-time equivalents. PPC teams offered direct, mainly medical and nursing care. Due to the shortage of qualified personnel, indirect care and support were either unavailable or quite limited. PPC services were offered 24/7 in some of the CG institutions and almost exclusively inside the hospital setting. The team’s composition, education and training of professionals and services corresponded to general PPC.^10^ In 2021 and 2022, the PPC teams cared for 79 patients and their families. See Figure 1 for a graphical representation of PPC *versus* SPPC.

### Data collection and management

All professionals who returned their informed consent forms received the questionnaire. The paper-pencil questionnaire, cover letter and pre-addressed return envelope were sent by mail to every study participant. Because all recipients had consented to participate, a response rate of 100% was expected. If questionnaires had not been returned 4 weeks after receipt, a reminder was sent *via* e-mail. If necessary, one more reminder was sent after two more weeks.

For each participant, pseudonymization was applied through the secuTrial^®^ data management system.^38^ All data that allowed the identification of the participants were stored separately on a secure server to which only three researchers from the main SPhAERA study team had access. The questionnaires were entered into the secuTrial electronic research forms by two study team members following an entry manual. Problems with data entry (e.g. if several answer categories were incorrectly ticked) were discussed within the study team, and solutions were documented in the entry manual.

### Measures

#### Work-related QoL

To assess work-related QoL, the German version of the Professional Quality of Life (ProQOL), fifth edition, instrument was used.^39^ The ProQOL covers the primary concepts of CS and CF. CF is further divided into BO and STS. The ProQOL instrument includes 30 items; one 10-item scale represents each of the three key concepts, that is, CS, BO and STS. Items are scored on a 5-point Likert-type scale ranging from 1 (never) to 5 (always). The ProQOL does not yield a single overall score.^27^ Each scale has a minimum score of 10 and a maximum score of 50; higher scores indicate higher levels of each concept. For each concept, a score of 22 or less is considered low, between 23 and 41 moderate and 42 or more high-level.^27^ According to the Concise ProQOL Manual, the alpha scale reliabilities are 0.88 for CS, 0.75 for BO and 0.81 for STS.^27^

#### Sociodemographic characteristics

Eleven sociodemographic variables were used: gender, age, living and family status, caring for children <18 years (yes, no) profession, palliative care training (formal, not formal), PPC training (yes, no), workplace, shift work (yes, no), employment workload (%) and work experience in pediatrics (years).

#### Potential confounders

To measure PPC and EoL exposure, the number of children professionals cared for during the last year in the palliative and EoL-phase was assessed with four categories: 0, 1–3, 4–6, >6.

The data collection took place at the end of the first and second years of the coronavirus disease 2019 (COVID-19) pandemic. The pandemic severely impacted all aspects of life, especially for professionals working in healthcare settings. Therefore, a question related to the COVID-19 pandemic was added to estimate the extent to which the pandemic influenced the professionals’ ProQOL ratings. The question ‘to what extent has the Corona situation influenced your answers about your work-related quality of life?’ was integrated and scored on an 11-point scale with endpoints of 0 (‘not at all’) and 10 (‘very much’).

### Data analysis

#### Aim 1: Measure work-related QoL in professionals not specialized in PPC

Descriptive statistics of central tendency and dispersion, percentages and frequencies were used to analyse sociodemographic variables. As specified in the official ProQOL manual, the three ProQOL scales were analysed separately.^27^ After reverse coding of negatively formulated items, raw sum scores and levels using the provided cut scores were calculated (levels: low* <* 22, moderate* >* 23/*<*41, high* >* 42).^27^ The Cronbach’s alphas for our sample were 0.70 in 2021 and 0.69 in 2022 for the BO scale, 0.71/0.76 for the STS scale and 0.84/0.85 for the CS scale.

A two-sample *t* test for continuous and Pearson’s Chi-square test for categorical variables was used to determine statistical differences between the IG and CG concerning the demographic variables. Further, the standardized mean difference (SMD) is reported. The SMD compares the difference between the IG and CG means. The SMD is not affected by sample size and provides a measure for the comparison of the relative balance of variables measured in different groups. An SMD of <0.1 is commonly considered negligible.^40^

#### Aim 2: Assess the difference in the work-related QoL in professionals not specialized in PPC with and without the availability of an SPPC team

Since participants’ characteristics differed substantially between the IG and CG, we used propensity score matching to estimate the intervention effect. Propensity scores were estimated using a generalized linear model with the following specifications: Nearest neighbour matching, full matching (multiple replacements) and a caliper of 0.2. The matching was performed with the R MatchIT package (version 4.5.1).^41^ The propensity score model included age, sex, profession, workplace, workload and shift work. The standard pair difference for the matched sample was 0.02. In addition to the variables included in the propensity score, the regression models to test the intervention effect were adjusted concerning training in PPC, number of children in PPC phase, number of children in EoL-phase and COVID-influence.

#### Aims 2 and 3: Explore which factors influence work-related QoL in professionals not specialized in PPC

A number of participants sent in responses for both years of data collection, creating dependencies (clustering) in our data. Therefore, a general estimation equation (GEE) model using participants as the clustering level was chosen. The ‘exchangeable’ correlation structure was selected. The model was fitted for each scale based on univariable analysis and theoretical considerations. The R geepack package version 1.3.9^42^ was used for the GEE model. All cases (i.e. all questionnaires from both years) were used to explore influential factors, since participants who participated either only once or only in 2021 did not differ statistically from those who participated twice or in 2022. Separate regression models were fitted to estimate the intervention’s effect and factors associated with the outcomes (BO, STS, CS). We conducted separate sensitivity analyses to check the models’ consistency when ‘year of participation’ was added and the ‘COVID-influence’ variable was omitted. Regression coefficients did not shift considerably between the differently specified regression models. For detailed results of the sensitivity analyses, see the Supplemental Material.

Missing data were analysed and checked regarding their distribution and randomness. Cases with missing data on any of the three scales were deleted for the respective analyses and propensity score matching. *p* Values of <0.05 were considered statistically significant. Statistical analyses were performed using R version 4.0.3.^43^

## Results

**Figure 2.**
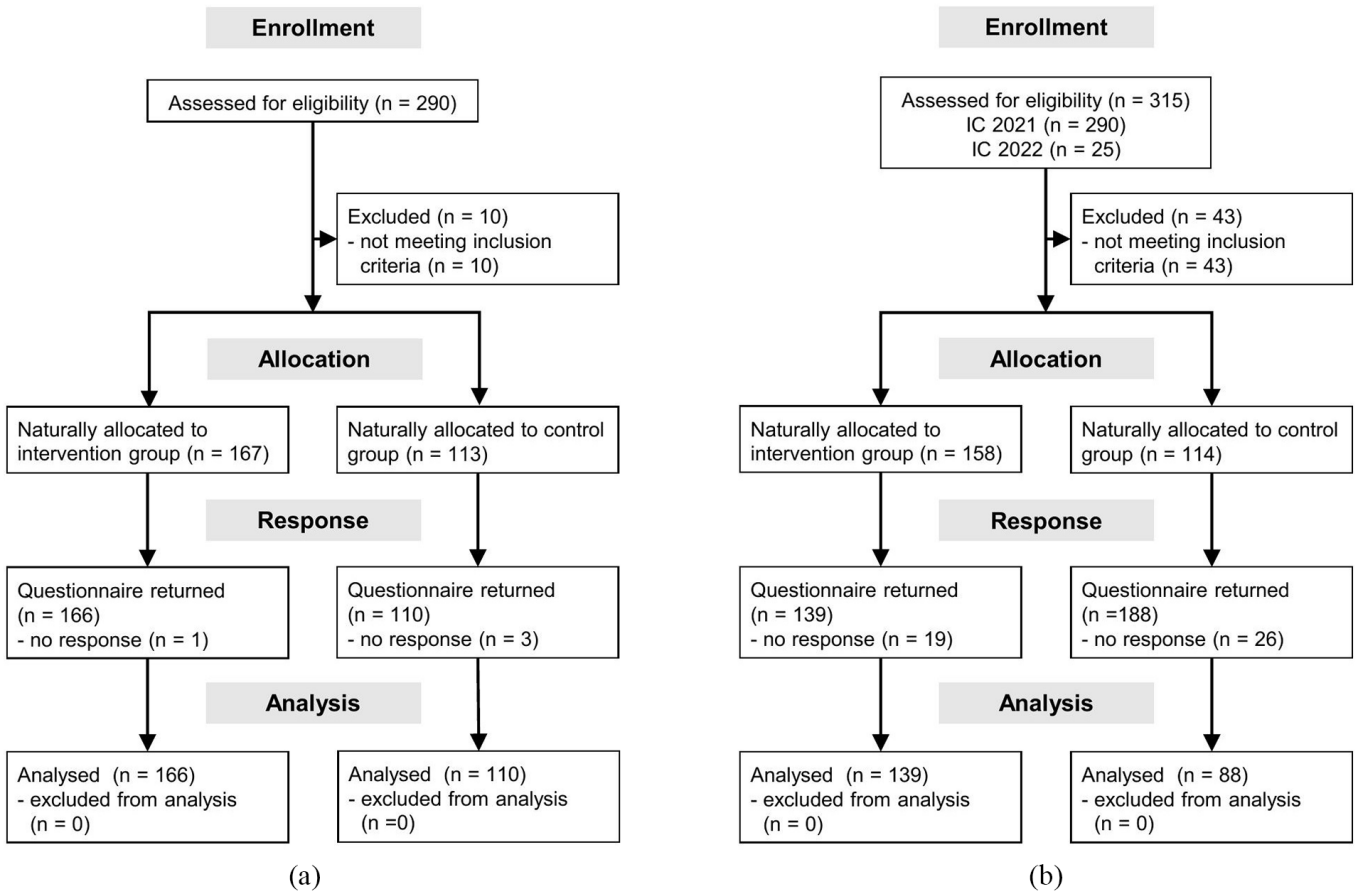
Flow diagram of participants for the years 2021 and 2022. (a) SPhAERA professionals – flow diagram 2021. (b) SPhAERA Professionals – flow diagram 2022. SPhAERA, Specialized Pediatric PAlliativE CaRe: Assessing family, healthcare professional and health system outcomes in a multi-site context of various care settings.

In 2021, of the 290 professionals who signed the informed consent form, 95% completed the questionnaire, that is, *n* = 276 participants for the 2021 data collection. In 2022, 88 participants found eligible in 2021 either no longer fulfilled the eligibility criteria, failed to return their questionnaires or could not be reached due to workplace changes. Their loss was offset by the entry of 25 additional eligible professionals who signed the informed consent and completed the questionnaire, resulting in a sample of 227 participants in 2022. Detailed information is displayed in the participants flow diagram (Figure 2). In total, 301 professionals participated across 2021 and 2022, with 202 participating in both years (67%), representing a total of 503 cases. Only a small proportion (maximum 5.2%) of values were missing. Participants’ characteristics (for all cases) are displayed in Table 1.

**Table 1.** Sociodemographic characteristics of participants (all cases).^a^.

Features/categories	Intervention (*n* = 305)	Comparison (*n* = 198)	*p* ^b^	SMD^b^
Gender, *n* (%)			**0.05**	**0.19**
Female	280 (92)	170 (86)		
Male	25 (8)	28 (14)		
Age (years)			0.7	0.04
Mean (SD)	42.73 (10.4)	43.09 (10.1)		
Profession, *n* (%)			**<0.01**	**0.4**
Registered nurse	164 (54)	140 (71)		
Physician	64 (21)	35 (18)		
Other professions	77 (25)	23 (11)		
Workplace, *n* (%)			**<0.01**	**0.62**
Hospital wards^c^	126 (41)	83 (42)		
PICU, NICU	89 (29)	48 (24)		
Homecare service, family practitioner	51 (17)	66 (33)		
Long-term care institutions	39 (13)	1 (1)		
Workload, *n* (%)			0.09	0.16
Full-time (⩾0.8)	188 (62)	106 (54)		
Part-time (⩽0.79)	117 (38)	92 (46)		
Shift work, *n* (%)	(*n* = 302)	(*n* = 194)	**0.01**	**0.26**
Yes	169 (56)	133 (69)		
No	133 (44)	61 (31)		
Pediatric work experience (years)	(*n* = 295)	(*n* = 196)	0.38	0.08
Mean (SD)	14.54 (8.98)	15.31 (10.02)		
Training in palliative care, *n* (%)	(*n* = 291)	(*n* = 195)	0.12	0.16
Formal	33 (11)	13 (7)		
No formal training	258 (89)	182 (93)		
Training in PPC, *n* (%)	(*n* = 296)		0.5	0.07
Yes	98 (33)	59 (30)		
No	198 (67)	139 (70)		
Living situation, *n* (%)		(*n* = 197)	0.59	0.09
With partner/children/other adults	234 (77)	150 (76)		
Alone	61 (20)	37 (19)		
Single parent	10 (3)	10 (5)		
Caring for children <18 years, *n* (%)	(*n* = 302)	(*n* = 194)	0.35	0.09
Yes	100 (33)	73 (38)		
No	202 (67)	121 (62)		

a ‘All cases’ signifies that participants who participated in both survey rounds (2021 and 2022) were counted twice.

b Significant *p* values (*p* value: <0.05) and corresponding SMDs have been written in bold.

c Hospital wards = Emergency department, medical/surgery/mixed wards, oncology and outpatient clinic.

NICU, neonatal intensive care unit; PICU, pediatric intensive care unit; PPC, pediatric palliative care; SD, standard deviation; SMD, standardized mean difference.

In the IG, 84% (*n* = 256) of professionals reported receiving support from the SPPC team, while 67% (*n* = 133) of those in the CG reported receiving support from the PPC team (*p* ⩽ 0.01; SMD = 0.41).

### Work-related QoL

**Figure 3.**
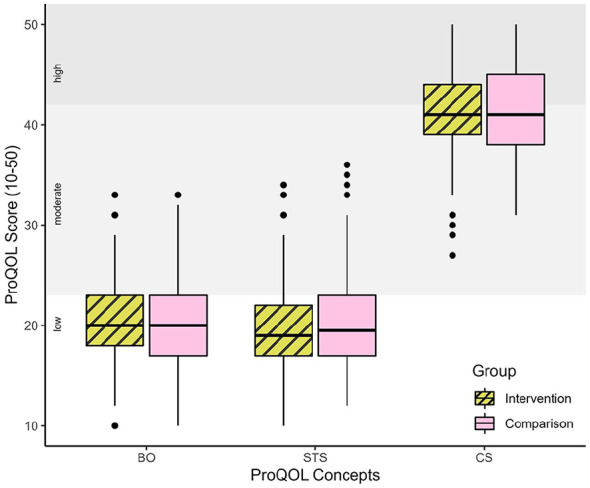
BO, STS and CS scores of professionals involved in PPC. BO, STS and CS scores for the intervention and comparison group are displayed as boxplots. The ProQOL score levels are embedded in the background and allow the classification of BO, STS and CS scores into the levels: low (score ⩽ 22), moderate (23 ⩽ score ⩾ 41) and high (score ⩾ 42). Participants who participated in both survey rounds (2021 and 2022) were counted twice. BO, burnout; CS, compassion satisfaction; PPC, pediatric palliative care; ProQOL, Professional Quality of Life; STS, secondary traumatic stress.

Figure 3 provides an overview of the results of the three ProQOL concepts.

#### Effectiveness of SPPC

Characteristics of matched participants (cases) are displayed as Supplemental Material. Before matching, IG and CG differed significantly regarding gender, profession, workplace and shift work. The matching process reduced all substantial differences except workplace.

The SPPC intervention showed no statistically significant effects on any of the three outcomes (BO, STS and CS). Compared to the CG, the IG had higher BO scores (1.09; *p* = 0.27), lower STS scores (−0.32; *p* = 0.74) and higher CS scores (−0.57; *p* = 0.48). The complete results of the unadjusted and adjusted models are shown in Table 2.

**Table 2.** Intervention effect – estimates of the GEE model for the scales BO, STS and CS using a matched sample (*n* = 357).

Variables	Levels	Unadjusted			Adjusted (full model)		
		Coefficient (SE)^a^	95% CI^a^	*p* Value^a^	Coefficient (SE)^a^	95% CI^a^	*p* Value^a^
BO							
Intervention variable: Group	Comparison (Ref)						
	Intervention	0.89 (1.04)	−1.15, 2.93	0.39	1.09 (0.98)	−0.83, 3.00	0.27
Training in PPC	No (Ref)						
	Yes	−0.23 (0.47)	−1.16, 0.69	0.62	−0.17 (0.42)	−0.99, 0.65	0.69
Number of children in PPC phase	1–3 (Ref)						
	>4	−0.55 (0.56)	−1.65, 0.55	0.33	−0.37 (0.55)	−1.46, 0.71	0.50
Number of children in EoL-phase	0 (Ref)						
	1–3	−0.40 (0.55)	−1.49, 0.68	0.47	−0.46 (0.45)	−1.34, 0.42	0.31
	>4	−0.94 (0.71)	−2.34, 0.46	0.19	−0.90 (0.77)	−2.41, 0.60	0.24
COVID-influence		**0.23 (0.11)**	**0.01, 0.45**	**0.04**	**0.25 (0.09)**	**0.07, 0.42**	**0.006**
STS							
Intervention variable: Group	Comparison (Ref)						
	Intervention	−0.42 (1.06)	−2.50, 1.65	0.69	−0.32 (0.96)	−2.19, 1.56	0.74
Training in PPC	No (Ref)						
	Yes	−0.21 (0.56)	−1.31, 0.88	0.70	−0.06 (0.50)	−1.04, 0.91	0.90
Number of children in PPC phase	1–3 (Ref)						
	>4	−0.78 (0.50)	−1.76, 0.20	0.12	−0.91 (0.53)	−1.96, 0.13	0.09
Number of children in EoL-phase	0 (Ref)						
	1–3	0.06 (0.58)	−1.07, 1.20	0.92	0.20 (0.56)	−0.90, 1.30	0.72
	>4	−0.46 (0.78)	−2.00, 1.08	0.56	0.02 (0.79)	−1.52, 1.56	0.98
COVID-influence		**0.27 (0.10)**	**0.07, 0.47**	**0.009**	**0.27 (0.09)**	**0.09, 0.44**	**0.003**
CS							
Intervention variable: Group	Comparison (Ref)						
	Intervention	−0.45 (0.82)	−2.07, 1.16	0.58	−0.57 (0.79)	−2.12, 0.99	0.48
Training in PPC	No (Ref)						
	Yes	−5.35e-03 (0.39)	−0.78, 0.77	0.99	−0.03 (0.38)	−0.78, 0.71	0.93
Number of children in PPC phase	1–3 (Ref)						
	>4	0.62 (0.47)	−0.31, 1.54	0.19	0.38 (0.50)	−0.60, 1.36	0.45
Number of children in EoL-phase	0 (Ref)						
	1–3	0.64 (0.46)	−0.26, 1.54	0.17	0.61 (0.46)	−0.29, 1.51	0.18
	>4	1.17 (0.64)	−0.09, 2.44	0.07	1.06 (0.68)	−0.28, 2.40	0.12
COVID-influence		−0.11 (0.09)	−0.28, 0.06	0.19	−0.13 (0.08)	−0.28, 0.02	0.08

a To facilitate reading, the significant coefficients, 95% CI and *p* values (*p* value: <0.05) have been written in bold.

95% CI, 95% confidence interval; BO, burnout; COVID, coronavirus disease; CS, compassion satisfaction; EoL, end of life; GEE, general estimation equation; PPC, pediatric palliative care; Ref, reference; SE, standard error; STS, secondary traumatic stress.

#### Influencing factors of work-related QoL

Several factors significantly influenced BO, STS and CS levels in the adjusted analysis. Compared to nurses, physicians had higher levels of BO (1.70; *p* = 0.02) and STS (2.69; *p* ⩽ 0.01) and lower scores on the CS scale; however, the latter differences were not statistically significant (−0.40; *p* = 0.62). Additionally, men had significantly lower STS scores than women (−2.34, *p* ⩽ 0.01). Also, the COVID-19 pandemic significantly influenced the participants’ ratings on all three ProQOL scales. The higher they rated the influence of the pandemic on their ProQOL rating, the higher their BO and STS levels (BO 0.29, *p* ⩽ 0.001; STS 0.23, *p* ⩽ 0.001) and the lower their levels of CS (CS −0.13, *p* ⩽ 0.01). See Tables 3 to 5 for the detailed results of each GEE model for BO, STS and CS.

**Table 3.** Influencing factors of work-related QoL–GEE estimates of the regression model for the scale burnout (*n* = 459).

Varaibles	Levels	Unadjusted			Adjusted (full model)		
		Coefficient (SE)^a^	95% CI^a^	*p* Value^a^	Coefficient (SE)^a^	95% CI^a^	*p* Value^a^
Predictors							
Age		−0.04 (0.02)	−0.08, 0.00	0.08	−7.09e-03 (0.02)	−0.05, 0.03	0.73
Sex	Female (Ref)						
	Male	0.18 (0.63)	−1.05, 1.42	0.77	−0.95 (0.65)	−2.23, 0.33	0.15
Profession	Nurses (Ref)						
	Physicians	1.07 (0.57)	−0.04, 2.19	0.06	**1.70 (0.71)**	**0.31, 3.09**	**0.02**
	Others	0.20 (0.56)	−0.89, 1.30	0.71	0.51 (0.67)	−0.79, 1.82	0.44
Workplace	Hospital wards (Ref)						
	PICU, NICU	0.01 (0.59)	−1.15, 1.16	0.99	0.16 (0.58)	−0.97, 1.30	0.78
	Homecare	−0.86 (0.56)	−1.95, 0.24	0.13	−0.22 (0.57)	−1.33, 0.89	0.70
	Long-term care inst.	0.20 (0.76)	−1.30, 1.69	0.80	−0.21 (0.77)	−1.72, 1.31	0.789
Workload	Part-time (<0.79) (Ref)						
	Full-time (>0.8)	**0.93 (0.39)**	**0.16, 1.70**	**0.02**	0.61 (0.42)	−0.22, 1.43	0.15
Shiftwork	No (Ref)						
	Yes	0.21 (0.42)	−0.61, 1.04	0.61	0.49 (0.50)	−0.50, 1.47	0.33
Training in PPC	No (Ref)						
	Yes	−0.44 (0.31)	−1.05, 0.18	0.16	−0.30 (0.31)	−0.91, 0.31	0.34
Number of children in PPC phase	1–3 (Ref)						
	>4	0.07 (0.37)	−0.65, 0.78	0.85	0.12 (0.40)	−0.67, 0.91	0.76
Number of children in EoL-phase	0 (Ref)						
	1–3	0.04 (0.35)	−0.64, 0.72	0.91	0.10 (0.37)	−0.63, 0.83	0.79
	>4	−0.71 (0.61)	−1.90, 0.49	0.25	−0.71 (0.68)	−2.04, 0.61	0.29
Confounder							
COVID-influence		**0.29 (0.06)**	**0.16, 0.42**	**<0.001**	**0.29 (0.06)**	**0.17, 0.42**	**<0.001**

a To facilitate reading, the significant coefficients, 95% CI and *p* values (*p* value: <0.05) have been written in bold.

95% CI, 95% confidence interval; COVID, coronavirus disease; EoL, end of life; GEE, general estimation equation; NICU, neonatal intensive care unit; PICU, pediatric intensive care unit; PPC, pediatric palliative care; QoL, quality of life; Ref, reference; SE, standard error.

**Table 4.** Influencing factors of work-related QoL–GEE estimates of the regression model for the scale secondary traumatic stress (*n* = 467).

Variables	Levels	Unadjusted			Adjusted (full model)		
		Coefficient (SE)^a^	95% CI^a^	*p* Value^a^	Coefficient (SE)^a^	95% CI^a^	*p* Value^a^
Predictors							
Age		0.00 (0.02)	−0.04, 0.05	0.90	0.02 (0.02)	−0.03, 0.06	0.46
Sex	Female (Ref)						
	Male	−0.47 (0.78)	−1.99, 1.05	0.55	**−2.34 (0.81)**	**−3.92, −0.76**	**0.004**
Profession	Nurses (Ref)						
	Physicians	**1.55 (0.63)**	**0.31, 2.79**	**0.02**	**2.69 (0.79)**	**1.15, 4.23**	**<0.001**
	Others	0.10 (0.56)	−1.00, 1.20	0.86	0.42 (0.69)	−0.93, 1.76	0.55
Workplace	Hospital wards (Ref)						
	PICU, NICU	0.15 (0.60)	−1.04, 1.33	0.81	0.57 (0.57)	−0.55, 1.68	0.32
	Homecare	−0.03 (0.69)	−1.38, 1.32	0.97	0.55 (0.74)	−0.90, 2.00	0.46
	Long-term care inst.	0.12 (0.81)	−1.47, 1.70	0.89	0.10 (0.84)	−1.54, 1.75	0.90
Workload	Part-time (<0.79) (Ref)						
	Full-time (>0.8)	**0.78 (0.39)**	**0.02, 1.53**	**0.04**	0.68 (0.38)	−0.08, 1.43	0.08
Shiftwork	No (Ref)						
	Yes	−0.05 (0.42)	−0.88, 0.77	0.90	0.22 (0.49)	−0.74, 1.17	0.66
Training in PPC	No (Ref)						
	Yes	−0.29 (0.36)	−0.99, 0.41	0.41	−0.25 (0.37)	−0.98, 0.47	0.50
Number of children in PPC phase	1–3 (Ref)						
	>4	−0.42 (0.35)	−1.10, 0.25	0.22	−0.49 (0.38)	−1.23, 0.25	0.19
Number of children in EoL-phase	0 (Ref)						
	1–3	0.00 (0.36)	−0.70, 0.69	0.99	0.31 (0.41)	−0.49, 1.11	0.45
	>4	−0.19 (0.62)	−1.40, 1.02	0.76	0.20 (0.63)	−1.04, 1.43	0.75
Confounder							
COVID-influence		**0.21 (0.06)**	**0.09, 0.33**	**<0.001**	**0.23 (0.06)**	**0.11, 0.35**	**<0.001**

a To facilitate reading, the significant coefficients, 95% CI and *p* values (*p* value: <0.05) have been written in bold.

95% CI, 95% confidence interval; COVID, coronavirus disease; EoL, end of life; GEE, general estimation equation; NICU, neonatal intensive care unit; PICU, pediatric intensive care unit; PPC, pediatric palliative care; QoL, quality of life; Ref, reference; SE, standard error.

**Table 5.** Influencing factors of work-related QoL–GEE estimates of the regression model for the scale compassion satisfaction (*n* = 472).

Variables	Levels	Unadjusted			Adjusted (full model)		
		Coefficient (SE)^a^	95% CI^a^	*p* Value^a^	Coefficient (SE)^a^	95% CI^a^	*p* Value^a^
Predictors							
Age		0.04 (0.02)	0.00, 0.08	0.051	0.02 (0.02)	−0.02, 0.07	0.27
Sex	Female (Ref)						
	Male	−0.10 (0.64)	−1.35, 1.15	0.87	0.15 (0.72)	−1.27, 1.57	0.84
Profession	Nurses (Ref)						
	Physicians	−0.43 (0.63)	−1.67, 0.80	0.49	−0.40 (0.80)	−1.97, 1.17	0.62
	Others	−0.06 (0.59)	−1.22, 1.10	0.92	0.20 (0.76)	−1.29, 1.68	0.80
Workplace	Hospital wards (Ref)						
	PICU, NICU	−0.15 (0.57)	−1.27, 0.96	0.79	−0.33 (0.58)	−1.47, 0.81	0.57
	Homecare	1.20 (0.65)	−0.07, 2.48	0.06	0.97 (0.74)	−0.47, 2.42	0.19
	Long-term care inst.	0.20 (0.71)	−1.19, 1.60	0.78	0.35 (0.76)	−1.13, 1.84	0.64
Workload	Part-time (<0.79) (Ref)						
	Full-time (>0.8)	−0.48 (0.44)	−1.34, 0.37	0.27	−0.21 (0.48)	−1.16, 0.73	0.66
Shiftwork	No (Ref)						
	Yes	−0.12 (0.44)	−0.98, 0.75	0.79	−0.03 (0.53)	−1.07, 1.02	0.96
Training in PPC	No (Ref)						
	Yes	−0.04 (0.31)	−0.64, 0.56	0.90	−0.24 (0.32)	−0.86, 0.38	0.45
Number of children in PPC phase	1–3 (Ref)						
	>4	0.05 (0.35)	−0.63, 0.73	0.89	−0.02 (0.41)	−0.82, 0.78	0.96
Number of children in EoL-phase	0 (Ref)						
	1–3	0.11 (0.35)	−0.58, 0.80	0.76	0.09 (0.40)	−0.69, 0.87	0.82
	>4	0.89 (0.51)	−0.11, 1.90	0.08	0.93 (0.58)	−0.20, 2.06	0.11
Confounder							
COVID-influence		−0.14 (0.06)	−0.25, −0.03	**0.02**	−0.13 (0.06)	−0.25, −0.02	**0.02**

a To facilitate reading, the significant coefficients, 95% CI and *p* values (*p* value: <0.05) have been written in bold.

95% CI, 95% confidence interval; COVID, coronavirus disease; EoL, end of life; GEE, general estimation equation; NICU, neonatal intensive care unit; PICU, pediatric intensive care unit; PPC, pediatric palliative care; QoL, quality of life; Ref, reference; SE, standard error.

## Discussion

This repeated cross-sectional comparative effectiveness study shows overall low to moderate BO and STS levels and moderate to high CS levels in professionals without specialized training who work with children receiving palliative or EoL care and their families. None of our participants had high BO or STS, or low CS. On the contrary, the majority had high CS in combination with low or moderate BO and STS scores, signifying what Stamm called the ‘most positive result’.^27^ No statistical difference was observed between the IG and CG in levels of BO, STS or CS. Therefore, regarding our IG of professionals who are not specialized in PPC, but who care for children through PPC or EoL phases, the impact of an SPPC team’s availability on that group’s work-related QoL remains unclear. Profession (physicians), gender (male) and the COVID-19 pandemic were found to be influential for our sample’s levels of BO, STS or CS.

Our assumption that professionals in the IG would report a higher work-related QoL than those in the CG did not hold. Similarly, in a pre-post intervention study, Brandon *et al.*^44^ could not demonstrate any impact of an SPPC team on healthcare professionals’ distress levels. Consequently, we must reflect critically on whether work-related QoL is the most suitable outcome for assessing the effectiveness of an SPPC team’s input at the professional level. As a highly complex construct, QoL is extremely challenging to operationalize. This is likely why an intervention targeting the support and training of professionals working in an emotionally challenging setting showed only a marginal impact on work-related QoL.

Supporting this position, Stamm acknowledges the complexity of work-related QoL and highlights work environment characteristics that influence work-related QoL.^27^ Similarly, in a Delphi study, Maassen *et al.*^45^ identified 36 elements that are important for a positive work environment. These elements range from interpersonal factors, for example, supportive managers, engaged leadership, healthcare professionals’ autonomy, supportive co-workers and teamwork, to available structural and electronic resources.^45^ None of these elements are clearly represented by the current version 5 of the ProQOL, which limits the instrument conceptually.

The ProQOL scales are widely used to measure the concepts of BO, STS and CS in professionals.^46,47^ Nevertheless, both Hemsworth *et al.*^46^ and Geoffrion *et al.*^47^ have criticized the ProQOL instrument and its properties and proposed adapting the scales to enhance their construct validity and reliability. And when Hotchkiss and Wong^48^ used a systematic meta-analysis to explore the ProQOL’s factorial structure across various settings, cultures and languages, they found that CS is a reliable and valid construct. However, the strong mean correlation between BO and STS indicated that their relationship within the 30-item ProQOL is too close, making a distinct interpretation difficult. Overall, the meta-analysis found that the STS and BO scales need revision.^48^

Quite frankly, considering the current problems health systems face, this sample’s low/moderate BO and STS and moderate/high CS levels are astonishing. To our knowledge, only a few studies have focussed specifically on work-related QoL in professionals working in PPC. Using the Compassion Fatigue and Satisfaction Self-Test for Helpers (CFST), Kase *et al*.^29^ found mean scores of 17.9 for CF (scale range: 0–90), 17.6 for BO (scale range: 0–65) and 89.7 for CS (scale range: 0–115). Based on predefined cutoff points they concluded that 18% of their sample experienced CF, 12% BO and 25% CS.^29^ A recent national survey of staff well-being in UK children’s hospices found that 89% of the 518 participants showed low to intermediate levels of BO, resulting in a BO prevalence of 11%, as measured by the Copenhagen Burnout Inventory.^49^ In pediatric (but not specifically PPC) settings, another study found satisfactory work-related QoL in 173 pediatric nurses, two-thirds of whom worked in intensive care units.^50^ In line with our findings, low STS and satisfactory CS levels measured with the CFST were found.

Other studies that focused on the pediatric setting had contrasting results. Using a modified CFST in pediatric emergency medicine physicians, Gribben *et al.*^51^ found that 16% were at risk for CF and 22% for BO. Berger *et al.*^52^ reported high BO in 29%, high STS in 27% and low CS in 29% of pediatric nurses in a hospital setting. In a study using ProQOL data from a sample of 268 pediatric licensed nurses, advanced practice nurses and nurse leaders working on a hospital campus, Walden *et al.*^53^ found that 51% had high levels and 49% low levels of BO.^53^ Conversely, in the same study, STS and CS levels aligned with our findings, with low to moderate STS levels and medium to high CS levels.

Physicians in our study had higher BO and STS levels than nurses. This is contrary to the findings both of Bowden *et al.*^18^ and Kase *et al*.,^29^ neither of whom found any significant differences between professions. However, in line with our results, BO has been described as a ‘serious health care challenge’^54^ affecting physicians at ‘epidemic levels’.^55^ Further, studies using the Maslach Burnout Inventory have found that approximately half of pediatric physicians, fellows or residents reported high levels of BO or met the threshold for BO.^56–59^ Interestingly, the physicians in our sample also reported higher CS values. This seeming incongruity is explained in other studies describing how work can be simultaneously rewarding and burdensome.^18,25,26^ Larsen and Stamm^60^ explain the combination of high satisfaction with signs of CF and BO as a protective factor: ‘ (. . .) simultaneously embracing the benefits of the work, while experiencing the negative costs’, suggesting that ‘CS may be the most potent force in motivating continued work even in the presence of the “costs” of caring’ (p. 283).

Concerning STS, lower levels were found in males than in females. In a systematic review, Baum^61^ explored gender differences in susceptibility to STS among professionals working with traumatized clients. Supporting our finding, when assessing STS in relation to post-traumatic stress disorder symptomatology, females were more susceptible to STS compared to male professionals.^61^ Four of the 10 included studies assessed STS through the ProQOL instruments. Greater female susceptibility was reported in two studies, higher male susceptibility in one; and one study reported no gender difference concerning STS levels in professionals.^61^ Study findings are inconsistent and our female/male distribution was severely skewed towards females. Therefore, gender differences concerning STS and overall work-related QoL should be further investigated.

Explanations for the high CS score in combination with low or moderate BO and STS in our sample may be the professional experience of our study participants, as our sample consisted of older, highly experienced professionals. Participants had an average work experience of 15 years, with a mean age of 43 years. However, it remains unclear whether mainly experienced older professionals are assigned to PPC and EoL patients, meaning that younger, less experienced professionals are less exposed to these situations, or if, in the participating hospitals, younger professionals receive similar exposure to these situations but did not participate in this study. Simultaneously, our sample’s low levels of BO and STS may be explained by what Wang *et al.*^62^ named ‘survivor bias’ in their study of palliative care physicians. That is, as persons prone to BO and stress tend to leave the workforce,^62^ professionals who become highly experienced can only do so by developing effective coping mechanisms and resilience. Despite this, Jones *et al.*^63^ noted that more experienced professionals felt more confident but not more comfortable in PPC: ‘No matter how long practitioners engage in PPC, it continues to be difficult and emotionally challenging’ (p. 53).

Although no participating professionals indicated high levels of BO/STS or low CS, 27% had moderate BO, 25% moderate STS and 50% moderate CS. BO, STS and CS have been linked to intention to leave the workforce and staff turnover. In a sample of 10,163 female nurses, Pang *et al*.^64^ evaluated the effects of depressive symptoms and work-related QoL on their turnover intention. When STS levels were moderate or high, they found that the chance for turnover intention increased by 1.14; and with similar levels of BO, it increased by 1.54. Concerning CS, moderate or high levels decreased turnover intention by 0.72 and 0.52 times.^64^ Prost and Middleton^65^ found relationships between intention to leave and all ProQOL concepts in child welfare professionals. Considering that turnover and workforce shortages are a global healthcare issue,^66^ and that staff recruitment and retention are high priorities in healthcare institutions,^67^ attention must also be paid to moderate levels of BO, STS and CS, as these can function as predictors of intention to leave.

Inadvertently, we conducted our study during the first 2 years of the COVID-19 pandemic. Our self-developed item on the pandemic’s influence on the participants reporting on their work-related QoL was consistently the single most influential factor regarding BO, STS and CS. Kase *et al.*^68^ compared pre- and early-pandemic BO, STS and CS levels in several pediatric subspecialists and found no statistically significant differences. In contrast, a study aimed at defining how the COVID-19 pandemic has impacted PPC and EoL care found that through its profound impact on care provision high incidence of moral distress was reported among the 207 participating PPC team members. And while it is obvious that the pandemic years left their marks on everyone, these marks were particularly strong in healthcare professionals. However, it is still too early to say how the pandemic will affect healthcare staffing projections over the coming years.

## Strengths and limitations

To interpret this paper’s results, certain strengths and weaknesses need to be considered. One notable strength was our use of a complex scientific approach that applied a comparative effectiveness design to assess the impact of an SPPC program in a real-world setting. Another strength was the inclusion of professionals from different professional backgrounds and disciplines, which allowed further exploration of work-related QoL. Furthermore, we drew our sample from a range of settings in which PPC and EoL care is provided.

Considering the study’s timing – during the first 2 years of the COVID-19 pandemic – the inclusion of a specific question on the pandemic’s influence is another of this study’s strengths. The responses provide insights about the pandemic’s effects on the well-being of professionals in the PPC setting. The pandemic had far-reaching effects on the working conditions and workload of professionals. Challenging working conditions may have caused selection bias, as increased workloads led some professionals to decline study participation. Unfortunately, the actual study participation rate is unknown due to the applied recruitment approach. Further, our use of non-randomized participant groups led to significant differences between IG and CG, which could not be fully resolved through propensity score matching. And finally, as only observed factors could be included in our controlling approach, unobserved factors remained uncontrolled for. As a result, our results do not allow causal inferences.

## Conclusion

PPC is an emerging medical discipline serving the growing population of children living with life-limiting conditions and their families. While professionals providing PPC and EoL care have expressed their need for additional SPPC team support, no difference was found in the work-related QoL of professionals who received SPPC support compared to those who received none. Therefore, the hypothesized positive effect of a consultative SPPC service model on work-related QoL in professionals caring for children in PPC or EoL phases could not be shown. In this study’s sample, all professionals had low to moderate levels of BO and STS and moderate to high levels of CS. However, compared to other professionals, physicians had higher BO and STS levels. Further research is needed to identify professionals at risk for low work-related QoL and to advance the SPPC intervention components. Similarly, providing tailored support to professionals working in PPC will require more evidence regarding the key SPPC support elements and their effectiveness.

Further research is necessary to develop and apply designs that are adequate to evaluate SPPC teams’ effectiveness. Perhaps most importantly, attention must be paid to the selection of appropriate and relevant outcomes, which might extend to areas such as training and capacity building and interprofessional partnerships. Concepts such as BO, STS and CS are central to the assessment of work-related QoL. However, they might be insufficient to explain a complex construct like work-related QoL in its entirety.

## Supplemental Material

sj-docx-1-pcr-10.1177_26323524241247857 – Supplemental material for Work-related quality of life in professionals involved in pediatric palliative care: a repeated cross-sectional comparative effectiveness study Supplemental material, sj-docx-1-pcr-10.1177_26323524241247857 for Work-related quality of life in professionals involved in pediatric palliative care: a repeated cross-sectional comparative effectiveness study by Anne-Kathrin Gerber, Ursula Feuz, Karin Zimmermann, Stefan Mitterer, Michael Simon, Nicolas von der Weid and Eva Bergsträsser in Palliative Care and Social Practice

sj-docx-2-pcr-10.1177_26323524241247857 – Supplemental material for Work-related quality of life in professionals involved in pediatric palliative care: a repeated cross-sectional comparative effectiveness study Supplemental material, sj-docx-2-pcr-10.1177_26323524241247857 for Work-related quality of life in professionals involved in pediatric palliative care: a repeated cross-sectional comparative effectiveness study by Anne-Kathrin Gerber, Ursula Feuz, Karin Zimmermann, Stefan Mitterer, Michael Simon, Nicolas von der Weid and Eva Bergsträsser in Palliative Care and Social Practice

## Declarations

**Ethics approval and consent to participate:** This study was conducted according to the guidelines of the Declaration of Helsinki and was approved by all responsible regional Swiss ethics commissions (BASEC Nr. 2019-01170). It is registered in ClinicalTrials.gov (NCT04236180). Informed consent was obtained from all subjects involved in the study.

**Consent for publication:** Not applicable.

**Availability of data and materials:** The data presented in this study are available on request from the corresponding author, but are not publicly available due to ethical and privacy-related concerns.
