# Supplementary material for: Work-related quality of life in professionals involved in pediatric palliative care: a repeated cross-sectional comparative effectiveness study
Source: Palliat Care Soc Pract. 2024 May 9;18:26323524241247857. doi: 10.1177/26323524241247857 (PMC11085006; doi:10.1177/26323524241247857)
Supplement: sj-docx-1-pcr-10.1177_26323524241247857 – Supplemental material for Work-related quality of life in professionals involved in pediatric palliative care: a repeated cross-sectional comparative effectiveness study [file sj-docx-1-pcr-10.1177_26323524241247857.docx]

**Supplemental material for “Work-related quality of life in professionals involved in paediatric palliative care – a repeated cross-sectional comparative effectiveness study”**

**Supplemental Table 1.** **Sensitivity Analysis** - Estimates of the GEE model for the subscales BO, STS and CS using a matched sample (n = 357), including year of participation, and excluding COVID-influence

|  |  | **Adjusted (full model including year)** | | | **Adjusted (full model excluding COVID influence)** | | |
| --- | --- | --- | --- | --- | --- | --- | --- |
|  |  | **Coefficient (SE)^a^** | **CI 95%^a^** | ***p*-value^a^** | **Coefficient (SE)^a^** | **CI 95%^a^** | ***p*-value^a^** |
| **Burnout** | | | | | | | |
| Intervention variable: **Group** | Comparison (Ref) |  |  |  |  |  |  |
|  | Intervention | 1.03 (0.93) | -0.80, 2.85 | 0.27 | 0.92 (0.99) | -1.01, 2.85 | 0.35 |
| Training in PPC | No (Ref) |  |  |  |  |  |  |
|  | Yes | -0.06 (0.44) | -0.92, 0.80 | 0.89 | -0.26 (0.42) | -1.09, 0.57 | 0.54 |
| Number of children in PPC phase | 1-3 (Ref) |  |  |  |  |  |  |
|  | >4 | -0.36 (0.55) | -1.44, 0.72 | 0.52 | -0.32 (0.56) | -1.41, 0.78 | 0.57 |
| Number of children in EoL phase | 0 (Ref) |  |  |  |  |  |  |
|  | 1-3 | -0.41 (0.44) | -1.28, 0.45 | 0.35 | -0.32 (0.51) | -1.31, 0.67 | 0.53 |
|  | >4 | -0.79 (0.78) | -2.31, 0.73 | 0.31 | -0.81 (0.75) | -2.27, 0.66 | 0.28 |
| COVID-influence |  | **0.23 (0.09)** | **0.05, 0.41** | **0.01** |  |  |  |
| Year | 2021 (Ref) |  |  |  |  |  |  |
|  | 2022 | 0.56 (0.34) | -0.12, 1.23 | 0.11 |  |  |  |
| **Secondary Traumatic Stress** | | | | | | | |
| Intervention variable: **Group** | Comparison (Ref) |  |  |  |  |  |  |
|  | Intervention | -0.36 (0.94) | -2.20, 1.48 | 0.70 | -0.50 (1.02) | -2.49, 1.49 | 0.62 |
| Training in PPC | No (Ref) |  |  |  |  |  |  |
|  | Yes | 0.02 (0.50) | -0.96, 0.99 | 0.97 | -0.16 (0.52) | -1.18, 0.86 | 0.76 |
| Number of children in PPC phase | 1-3 (Ref) |  |  |  |  |  |  |
|  | >4 | -0.90 (0.52) | -1.92, 0.12 | 0.08 | -0.88 (0.56) | -1.97, 0.21 | 0.12 |
| Number of children in EoL phase | 0 (Ref) |  |  |  |  |  |  |
|  | 1-3 | 0.23 (0.56) | -0.86, 1.33 | 0.68 | 0.36 (0.63) | -0.86, 1.59 | 0.56 |
|  | >4 | 0.11 (0.80) | -1.46, 1.67 | 0.90 | 0.15 (0.83) | -1.47, 1.77 | 0.86 |
| COVID-influence |  | **0.25 (0.09)** | **0.08, 0.43** | **0.004** |  |  |  |
| Year | 2021 (Ref) |  |  |  |  |  |  |
|  | 2022 | 0.38 (0.31) | -0.22, 0.99 | 0.21 |  |  |  |

|  |  | **Adjusted (full model including year)** | | | **Adjusted (full model excluding COVID influence)** | | |
| --- | --- | --- | --- | --- | --- | --- | --- |
|  |  | **Coefficient (SE)^a^** | **CI 95%^a^** | ***p*-value^a^** | **Coefficient (SE)^a^** | **CI 95%^a^** | ***p*-value^a^** |
| **Compassion Satisfaction** | | | | | | | |
| Intervention variable: **Group** | Comparison (Ref) |  |  |  |  |  |  |
|  | Intervention | -0.57 (0.79) | -2.12, 0.99 | 0.48 | -0.48 (0.79) | -2.02, 1.06 | 0.54 |
| Training in PPC | No (Ref) |  |  |  |  |  |  |
|  | Yes | -0.03 (0.39) | -0.79, 0.73 | 0.94 | 0.01 (0.38) | -0.73, 0.76 | 0.97 |
| Number of children in PPC phase | 1-3 (Ref) |  |  |  |  |  |  |
|  | >4 | 0.38 (0.50) | -0.60, 1.36 | 0.45 | 0.37 (0.51) | -0.63, 1.36 | 0.47 |
| Number of children in EoL phase | 0 (Ref) |  |  |  |  |  |  |
|  | 1-3 | 0.61 (0.46) | -0.28, 1.51 | 0.18 | 0.53 (0.48) | -0.42, 1.47 | 0.27 |
|  | >4 | 1.06 (0.69) | -0.29, 2.42 | 0.12 | 0.99 (0.70) | -0.38, 2.36 | 0.16 |
| COVID-influence |  | -0.13 (0.08) | -0.28, 0.02 | 0.08 |  |  |  |
| Year | 2021 (Ref) |  |  |  |  |  |  |
|  | 2022 | 0.02 (0.29) | -0.54, 0.59 | 0.93 |  |  |  |
| ^a^ To facilitate reading, the significant coefficients, CI 95%, and *p*-values (*p*-value: <0.05) have been written in bold.  CI 95% = 95% Confidence Interval; EoL = End of Life; PPC = Paediatric Palliative Care; Ref = Reference; SE=Standard Error | | | | | | | |

**Supplemental Table 2.** Demographic characteristics of matched participants (all cases)^a^

| **Features / Categories** | Intervention  (*n* = 265) | Comparison  (*n* = 92) | *p*^b^ | SMD^b^ |
| --- | --- | --- | --- | --- |
| **Gender, *n* (%)** |  |  | 0.74 | 0.07 |
| Female | 244 (92) | 83 (90) |  |  |
| Male | 21 (8) | 9 (10) |  |  |
| **Age (y)** |  |  | 0.64 | 0.06 |
| Mean (SD) | 42.51 (10.20) | 41.93 (9.24) |  |  |
| **Profession, *n* (%)** |  |  | 0.09 | 0.27 |
| Registered nurse | 141 (53) | 61 (66) |  |  |
| Physician | 60 (23) | 14 (15) |  |  |
| Other professions | 64 (24) | 17 (19) |  |  |
| **Workplace, *n* (%)** |  |  | **0.01** | **0.49** |
| Hospital wards^c^ | 113 (43) | 43 (47) |  |  |
| PICU, NICU | 78 (29) | 27 (29) |  |  |
| Homecare service, family practitioner | 41 (16) | 21 (23) |  |  |
| Long-term care institutions | 33 (12) | 1 (1) |  |  |
| **Workload, *n* (%)** |  |  | 0.47 | 0.1 |
| Full-time (>0.8) | 163 (62) | 52 (57) |  |  |
| Part-time (<0.79) | 102 (38) | 40 (43) |  |  |
| **Shift work, *n* (%)** |  |  | 0.25 | 0.15 |
| Yes | 147 (55) | 58 (63) |  |  |
| No | 118 (45) | 34 (37) |  |  |
| **Paediatric work experience (y)** |  |  | 0.88 | 0.02 |
| Mean (SD) | 14.56 (8.90) | 14.73 (9.27) |  |  |
| **Training in palliative care, n (%)** |  |  |  |  |
| Formal | 29 (11) | 4 (4) | 0.08 | 0.26 |
| No formal training | 230 (89) | 88 (96) |  |  |
| **Training in PPC, n (%)** |  |  |  |  |
| Yes | 86 (32) | 20 (22) | 0.07 | 0.24 |
| No | 179 (68) | 72 (78) |  |  |
| **Living situation, *n* (%)** |  |  |  |  |
| With partner/children/other adults | 203 (77) | 71 (77) | 0.62 | 0.11 |
| Alone | 53 (20) | 16 (17) |  |  |
| Single parent | 9 (3) | 5 (6) |  |  |
| **Caring for children <18 years, *n* (%)** |  |  |  |  |
| Yes | 91 (35) | 34 (37) | 0.78 | 0.05 |
| No | 172 (65) | 58 (63) |  |  |
| ^a^ “All cases” signifies that participants who participated in both survey rounds (2021 and 2022) were counted twice.  ^b^ Significant *p*-values (*p*-value: <0.05) and corresponding SMDs have been written in bold  ^c^ Hospital wards = Emergency Department, Medical/Surgery/Mixed wards, Oncology, and outpatient clinic  PICU = Paediatric Intensive Care Unit; PPC = Paediatric Palliative Care; NICU = Neonatal Intensive Care Unit; SD = Standard deviation; SMD = Standardised mean difference | | | | |
